# Draft genome of the caprolactam-degrading *Paenarthrobacter* sp. CAP02 isolated from a landfill

**DOI:** 10.1128/mra.00650-24

**Published:** 2024-08-29

**Authors:** David A. Rivera-Kohr, Damayanti Rodriguez-Ramos, Lewis Tanner, Phuong Vo, Neha A. Thalpur, Hans C. Nielsen-Fox, Fuad J. Shatara, Craig A. Bingman, Liyuan Hou, Brian G. Fox, Erica L.-W. Majumder

**Affiliations:** 1 Department of Biochemistry, University of Wisconsin–Madison, Madison, Wisconsin, USA; 2 Department of Bacteriology, University of Wisconsin–Madison, Madison, Wisconsin, USA; 3 Department of Civil and Environmental Engineering, Utah State University, Logan, Utah, USA; University of Maryland School of Medicine, Baltimore, Maryland, USA

**Keywords:** *Paenarthrobacter*, caprolactam, biodegradation, nylon

## Abstract

Here, we report the draft genome sequence of *Paenarthrobacter* sp. CAP02, which metabolizes caprolactam as a sole source of carbon and nitrogen. This bacterium was isolated from Dane County Landfill Site No. 2 in Madison, WI, USA.

## ANNOUNCEMENT

*Paenarthrobacter* is a genus of Gram-positive, obligate aerobic bacteria in the *Micrococcaceae* family that show promise for bioremediation applications due to their robust degradation of man-made pollutants like nylon manufacturing waste, petrochemicals, and pesticides (1–8). Caprolactam is the monomer of nylon-6, or polyamide-6 (PA6), one of the most common textile fibers. Unpolymerized caprolactam is a major byproduct of PA6 manufacturing that is often discarded into the environment in wastewater. Caprolactam is reported to have toxic effects on algae, plants, animals, and humans; therefore, its release into the environment is hazardous to ecosystems and public health (9–13). Discovering microbes and enzymes that degrade caprolactam can enable bioremediation and waste valorization applications to reduce harmful caprolactam pollution. In May 2021, waste and soil samples were collected from depths up to 80 feet in Dane County Landfill Site No. 2 in Madison, WI, USA, and transported to the Erica Majumder lab (University of Wisconsin–Madison). Microbes from these samples were enriched on various substrates at 30°C starting with undefined rich liquid medium (per liter: 5 g yeast extract, 10 g tryptone, 10 g NaCl, 5 g peptone, 5 g malt extract, 5 g glucose, and 10 g sucrose) containing a mixture of commercial polyethylene (Alfa Aesar), polystyrene (Sigma Aldrich), and PA6 (GoodFellow) powders (10 mg/mL each) and ending with liquid carbon-free basal medium (LCFBM) without NaCl and with 10 mg/mL caprolactam (Fisher Scientific) as the sole carbon and nitrogen source (14). The full passaging history can be found under BioSample SAMN41494577 (liquid cultures were shaken at 220 rpm starting with the second passage into PA6 extractables). At the end of the enrichment course, a single colony was inoculated into LCFBM with 10 mg/mL caprolactam as the sole carbon and nitrogen source. This culture was submitted for Small Shotgun Metagenome Sequencing (SeqCenter).

Genomic DNA was extracted using the ZymoBIOMICS DNA Miniprep Kit (Zymo Research). The sequencing library was prepared using the Illumina DNA Prep kit and sequenced in a NovaSeq X Plus sequencer (Illumina). A total of 11,527,107 pairs of reads (2 × 151 bp) were obtained, then analyzed using KBase (15). After read quality trimming with Trimmomatic (v0.36) (16), 11,369,484 pairs of reads remained. Using metaSPAdes (v3.15.3) (17), a total of 4,543,936 bp (377.8× genome coverage) were assembled into 24 contigs with *N*_50_ = 359,084 bp and GC% = 63.02. Genome quality was assessed using CheckM (v1.0.18) (18), which reported completeness of 99.71% and contamination of 0.78% using 31 reference genomes from the *Micrococcaceae* family, thus indicating an isolated *Micrococcaceae* bacterium. Genome annotation was performed using the NCBI Prokaryotic Genome Annotation Pipeline (v6.7) (19–21), which predicted 4,287 genes (98.0% protein-coding genes), including a complete set of 54 tRNA genes for all 20 canonical proteinogenic amino acids and all ribosomal RNA subunits (one 5S copy, one 16S copy, and one 23S copy). Whole-genome analysis against all available *Paenarthrobacter* genomes in GenBank (by 15 May 2024) using FastANI (v1.32) (22) showed that the closest type strains are *Paenarthrobacter* sp. AR02 (accession GCA_021538675.1) (98.1% Average Nucleotide Identity (ANI)) and *Paenarthrobacter* sp. UW852 (accession GCA_024608385.1) (97.0% ANI). All three of these *Paenarthrobacter* share >96.7% ANI with each other and 78%–92% ANI with all other *Paenarthrobacter*. With a suggested bacterial species demarcation criterion at 95%–96% ANI (23), we believe that these three strains may belong to a new *Paenarthrobacter* species.

## Data Availability

This Whole Genome Shotgun project has been deposited at DDBJ/ENA/GenBank under accession JBEEII000000000. The version described in this paper is version JBEEII010000000.1. The raw Illumina reads can be found under SRA run accession number SRR29224833. This genome project is indexed at GenBank under BioProject PRJNA1114757, and the microbe sample information can be found under BioSample SAMN41494577.
